# Topological Abnormalities of Functional Brain Network in Early-Stage Parkinson’s Disease Patients With Mild Cognitive Impairment

**DOI:** 10.3389/fnins.2020.616872

**Published:** 2020-12-21

**Authors:** Xiangbin Chen, Mengting Liu, Zhibing Wu, Hao Cheng

**Affiliations:** ^1^Department of TCM Internal Medicine, The First Affiliated Hospital of Guangzhou University of Traditional Chinese Medicine, Guangzhou, China; ^2^School of Music, Jimei University, Xiamen, China; ^3^Department of Ultrasonography, Shaanxi Cancer Hospital Affiliated to Xi’an Jiaotong University, Xi’an, China

**Keywords:** Parkinson’s disease, mild cognitive impairment, fMRI, graph theory, small world

## Abstract

Recent studies have demonstrated structural and functional alterations in Parkinson’s disease (PD) with mild cognitive impairment (MCI). However, the topological patterns of functional brain networks in newly diagnosed PD patients with MCI are unclear so far. In this study, we used functional magnetic resonance imaging (fMRI) and graph theory approaches to explore the functional brain network in 45 PD patients with MCI (PD-MCI), 22 PD patients without MCI (PD-nMCI), and 18 healthy controls (HC). We found that the PD-MCI, PD-nMCI, and HC groups exhibited a small-world architecture in the functional brain network. However, early-stage PD-MCI patients had decreased clustering coefficient, increased characteristic path length, and changed nodal centrality in the default mode network (DMN), control network (CN), somatomotor network (SMN), and visual network (VN), which might contribute to factors for MCI symptoms in PD patients. Our results demonstrated that PD-MCI patients were associated with disrupted topological organization in the functional network, thus providing a topological network insight into the role of information exchange in the underlying development of MCI symptoms in PD patients.

## Introduction

Parkinson’s disease (PD) is one of the most common neurodegenerative diseases with multiple movement disorders and non-motor symptoms. Among newly diagnosed PD patients, more than 20% will develop mild cognitive impairment (MCI) after 3–5 years. MCI is considered to be a high-risk factor for the further development of dementia, which will seriously affect the quality of patients’ lives (Kehagia et al., 2010). Unfortunately, the neural basis underlying the MCI in PD is still not well understood.

As one of the most promising neuroimaging methods, magnetic resonance imaging (MRI) involving voxel-based morphometry (VBM), diffusion tensor imaging (DTI), and functional magnetic resonance imaging (fMRI) has been widely used to explore the structural and functional abnormality of the brain in PD patients with MCI (PD-MCI). Evidence from VBM in PD-MCI showed structural atrophy in temporal, frontal, hippocampus, and thalamus regions, compared with PD without MCI (PD-nMCI) (Beyer et al., 2007; Chen et al., 2016; Gao et al., 2017). In a DTI study, FA values were found to be significantly decreased in parts of the corpus callosum in PD-MCI compared with healthy controls (HC) and no significant difference between patients with PD-nMCI and PD-MCI (Hattori et al., 2012). Another recent longitudinal DTI study showed significant mean diffusivity increase mainly in the frontal regions in the PD-MCI group when compared with the PD group with normal cognition (Minett et al., 2018). Moreover, investigators using the amplitude of low-frequency fluctuations (ALFF) and regional homogeneity (ReHo) as indicators in resting-state fMRI found PD patients with MCI had abnormal resting brain activity in the left middle temporal gyrus, right superior temporal gyrus, left superior frontal gyrus, right inferior frontal gyrus (Gao et al., 2016; Wang et al., 2018), left insula, and left precuneus (Li et al., 2020), compared with PD patients without MCI. The “structural atrophy” and “functional activity abnormalities” could indicate changes of neuronal plasticity (or due to synaptic loss), hyperexcitability, and neuronal circuit changes. The abnormalities in these regions were hypothesized to be the basis of neuroanatomy and pathophysiology in PD patients with MCI.

Recently, several studies by using resting-state fMRI found that functional disconnection could be also associated with MCI in PD. For example, the default mode network, which is highly relevant for cognitive processes, was found to have altered connectivity in PD with MCI (Hou et al., 2016). However, the other study showed that functional connectivity of the default mode network was altered in PD patients regardless of cognitive status, while a functional disconnection in the frontoparietal network was found to be associated with PD-MCI without detectable structural changes (Amboni et al., 2015). The patients “regardless of cognitive status” might indicate general pathological changes in the brain. MCI-related “topological changes in newly diagnosed PD” was mentioned but not directly supported by their results. Furthermore, findings in dynamic functional connectivity showed dynamic functional brain deterioration in PD-MCI, which is not present in PD without MCI (Díez-Cirarda et al., 2018). This evidence suggests that not only abnormalities in specific, discrete brain regions but also disruptions in functional connectivity or functional networks may be involved in the neural mechanisms of PD-MCI.

As an emerging method of network analysis, the graph theory modeled the brain as a complex functional system with topological features (such as small-world properties and nodal centralities), which are disrupted in PD patients. However, the MCI-related topological changes in the functional network were rarely explored, especially in the early-stage or newly diagnosed PD. Given the existence of structural and functional abnormalities in specific brain regions as well as disruption of functional connectivity in PD patients, it is plausible that the abnormalities of whole-brain topological networks in PD patients with MCI may be observed. Therefore, our study aimed to use resting-state fMRI data to find the MCI-related topological changes in newly diagnosed PD patients. First, we assessed the small-world topology of PD-nMCI, PD-MCI, and HC. Second, we investigated the topological parameters of the functional network (clustering coefficient, characteristic path length, and small-world index). Finally, we would like to evaluate the regions’ changes from the flow of information perspective among these three groups by using nodal centrality.

## Materials and Methods

### Participants

All MRI and experimental data used in this study were obtained from the Parkinson’s Progression Markers Initiative (PPMI)^1^, which is a large-scale, comprehensive observational, multicenter project of PD progression biomarkers (Marek et al., 2011). A total of 85 participants were analyzed, comprising 45 participants in the PD-nMCI group (mean age = 62.64 ± 9.86, 30 males), 22 in the PD-MCI group (mean age = 66.09 ± 8.56, 17 males), and 18 age- and sex-matched HC (mean age = 64.33 ± 9.87, 14 males) (Table 1). All PD patients were diagnosed according to the criteria of the United Kingdom Brain Bank (Hughes et al., 1992). The study was approved by Institutional Review Boards/Independent Ethics Committees. Written informed consent was obtained from all subjects. For more details on the study, please see http://www.ppmi-info.org/wp-content/uploads/2013/02/PPMI-Protocol-AM5-Final-27Nov2012v6-2.pdf.

**TABLE 1:** Demographic and clinical data of the subjects.

	PD-nMCI (*N* = 45)	PD-MCI (*N* = 22)	HC (*N* = 18)	Test factor	*P*-value
Age (years)	62.64 ± 9.86	66.09 ± 8.56	64.33 ± 9.87	*F* = 0.99	0.38
Gender (M/F)	30/15	17/5	14/4	χ^2^ = 1.23	0.54
Disease duration (years)	2.43 ± 1.22	2.78 ± 1.34	–	*T* = −1.07	0.06
UPDRS-III	20.49 ± 10.11	24.72 ± 12.49	–	*T* = −1.49	0.14
H&Y stage	1.69 ± 0.47	1.86 ± 0.35	–	*T* = −1.55	0.13
MoCa	28.24 ± 1.32	25.00 ± 2.99	27.56 ± 1.50	*F* = 21.34	<0.001
Education (years)	15.33 ± 2.84	15.86 ± 2.92	16.72 ± 2.67	*F* = 1.57	0.21

### MRI Data Acquisition

Imaging data were acquired on Siemens 3T MRI scanners. High-resolution structural images were acquired using a T1-weighted gradient-echo 3D MPRAGE sequence (TR = 2,300 ms, TE = 2.98, FA = 9°, 1 mm^3^ isotropic voxel). Resting-state fMRI scans were acquired with an echo-planar sequence (TR = 2,400 ms, TE = 25 ms, FA = 80°, voxel size = 3.3 mm^3^, total of 210 volumes, 40 axial slices). Subjects were advised to relax quietly with their eyes open for the resting-state functional scans while trying not to fall asleep.

### Data Preprocessing

The preprocessing workflow was performed using fMRIPrep 1.4.1 (Esteban et al., 2019), which is based on Nipype 1.2.0 (Gorgolewski et al., 2011) (details of the preprocessing process are provided in the Supplementary Material).

### Regions of Interest Parcelation

In the current study, we used the Schaefer parcelation template (Schaefer et al., 2018) with 100 parcels, each of which is related with one of the brain networks from the Yeo seven-network parcelation (Thomas Yeo et al., 2011)–the visual network (VN), dorsal attention network (DAN), somatomotor network (SMN), default mode network (DMN), limbic network (LN), frontoparietal task control network (CN), and ventral attention network (VAN) (Supplementary Table 2).

### Graph Theory Analysis of the Functional Brain Network

We transformed the matrix of the inter-regional correlation coefficient into a binary matrix: if the positive value of the correlation coefficient was larger than a certain threshold, there was a relationship (assigned “1”) in the matrix of 100 (occasionally 100 correlation coefficient); otherwise, there was no relationship (assigned “0”). There is currently no consensus among researchers on how to choose a fixed threshold. So, we threshed each matrix of correlation over a wide range of density (10 to 50% with an increase of 1%), then we estimated the properties of the resulting graphs at each threshold value. It can also describe the network with a continuous weighting between nodes, but this will result in complicated statistical feature descriptions in the graph theoretical analysis (He et al., 2007). This research, therefore, used binarized networks for explanations of statistical characteristics, which are comparatively simpler.

The coefficient *C* of the cluster parameter represents the complexity of network clustering (Watts et al., 1998; Sporns et al., 2004). The shortest path length indicated the shortest path for the information from one node to another node in the network. System resources were saved while information was transmitted more quickly through the shortest possible path (Latora and Marchiori, 2001). Small-world networks combine the benefits of regular networks (with a larger cluster coefficient and a longer characteristic path length) and random networks (with a smaller cluster coefficient and a shorter characteristic path length), which ensure the local and global efficiency of information transmission (Watts et al., 1998). A small-world index (σ = γ/λ) was used to measure the “small-world” characteristics of the network (Achard, 2006; Humphries et al., 2006). The betweenness centrality from a perspective of information flow describes the centrality of nodes (Freeman, 1977). We used *b_i_* = *B*(*i*)/*B* to normalize *B*(*i*), where *B* represents the mean betweenness centrality of all the nodes in the network (Melie-García et al., 2013). Then, we calculated the area under the curve (AUC) for each network to seek the group differences of *b*_*i*_, which offers a more straightforward scalar for brain network topology than the single threshold selection.

### Statistical Analysis

To determine if significant group differences existed in the parameters of the graph theory (cluster coefficient, characteristic path length, and nodal centrality), non-parametric permutation tests were performed between groups. In short, we first calculated the difference between groups in the average value of each parameter to test the null hypothesis for each parameter that the observed group difference could happen by chance. We then reassigned all the values into two groups randomly and recomputed the mean differences between the two randomized groups. This randomization procedure was repeated 10,000 times, resulting in distributions of differences between groups for each parameter. Finally, we used the 95% points of the distributions (two-tailed) as the confidence intervals to test the null hypothesis. If the null hypothesis was rejected (outside the confidence intervals), the differences of parameters in the functional brain network were thought to be significant.

## Results

### The Small-World Topology of Functional Brain Network in PD-MCI, PD-nMCI, and HC

**FIGURE 1:**
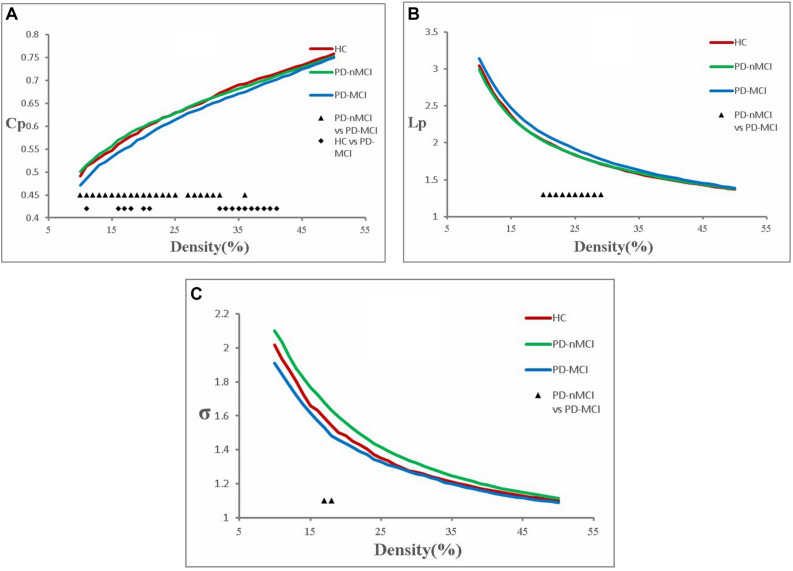
Cluster coefficient (Cp), characteristic path length (Lp), and small-world index (σ) in PD-MCI, PD-nMCI, and HC. **(A)** The Cp from the three groups. The black triangle means significant differences between PD-nMCI and PD-MCI (density thresholds: 10–25%, 27–32%, 36%, *P* < 0.05, two-tailed). The black prismatic means significant differences between HC and PD-MCI (density thresholds: 11%, 16–18%, 20–21%, 32–41%, *P* < 0.05, two-tailed). **(B)** The Lp from the three groups. The black triangle means significant differences between PD-nMCI and PD-MCI (density thresholds: 20–29%, *P* < 0.05, two-tailed). **(C)** The σ from the three groups. The black triangle means significant differences between PD-nMCI and PD-MCI (density thresholds: 17–18%, *P* < 0.05, two-tailed).

The functional brain networks in all the three groups had the characteristics of “small-world” networks. The small-world index of these three groups was larger than one (σ > 1) over an entire range of density thresholds, indicating that even in human brains afflicted with MCI, a relatively efficient network was needed to maintain the daily activities (Figure 1C).

### PD-MCI Patients versus PD-nMCI Patients (*P* < 0.05, Two-Tailed)

Compared with PD-nMCI patients, the PD-MCI patients showed significantly decreased clustering coefficient Cp (density thresholds: 10–25%, 27–32%, 36%) (Figure 1A), small-world index σ (density thresholds: 17 and 18%) (Figure 1C), and increased characteristic path length Lp (density thresholds: 20–29%) (Figure 1B). Meanwhile, increased nodal centrality in the VN, DMN, and CN was observed in the PD-MCI group, while there was decreased nodal centrality in the SMN (Table 2).

**TABLE 2:** Nodal centrality differences between groups.

	ROI label	ROI name	ROI network	*P*-value
PD-MCI > PD-nMCI	3	7Networks_LH_Vis_3	VN	0.037
	41	7Networks_LH_Default_Par_2	DMN	0.002
	88	7Networks_RH_Cont_PFCmp_1	CN	0.042
PD-nMCI > PD-MCI	60	7Networks_RH_SomMot_2	SMN	0.027

*ROI, region of interest.*

### PD-MCI Patients Versus the HC Group (*P* < 0.05, Two-Tailed)

Compared with the HC group, PD-MCI patients showed significantly decreased clustering coefficient Cp (density thresholds: 11%, 16–18%, 20–21%, 32–41%) (Figure 1A).

### PD-nMCI Patients versus the HC Group (*P* < 0.05, Two-Tailed)

There was no significant difference in Cp, Lp, and σ between the PD-nMCI and HC groups.

## Discussion

We employed an fMRI approach to seek the differences among PD-nMCI, PD-MCI, and HC to evaluate the topological changes of brain functional networks in early-stage PD patients with depression. Our results showed that compared with PD-nMCI, early-stage PD-MCI patients had decreased clustering coefficient, small-world index, and increased characteristic path length. Compared with the HC group, the PD-MCI groups showed a significantly decreased clustering coefficient. There was no significant difference in Cp, Lp, and σ between the PD-nMCI and HC groups. Therefore, we used nodal centrality to further test the hypothesis that small-world topology changes in PD-MCI patients may be accompanied by information communication alteration. Then, we found that nodal centrality was significantly increased in the VN, DMN, and CN, but significantly decreased in the SMN in PD-MCI compared with PD-nMCI. Due to the aim of seeking MCI-related changes in PD patients, we mainly focus on discussing the differences of topological organization between PD-MCI and PD-nMCI.

We found that the brain functional network of PD-MCI, PD-nMCI, and HC had a small-world property, which was consistent with many studies by using resting-state fMRI data in PD patients (Luo et al., 2015; Sang et al., 2015; Berman et al., 2016; Fang et al., 2017; Hou et al., 2020). Especially, in many studies of MCI and even dementia patients, the functional network still satisfies the network characteristics of the small world (Liu et al., 2012, 2014; Bai et al., 2013; Brier et al., 2014). Unlike a random network or regular network, a small-world network was found to be an optimized network for information separation and integration (Bullmore and Sporns, 2009). The brain functional network of PD-MCI and PD-nMCI patients also showed a small-world property in the current study, suggesting that even in patients with neurological and psychotic disorders (such as Alzheimer’s disease, PD, and MCI), a relatively efficient network was needed to maintain the daily activities.

Although the functional brain networks of these three groups retain small-world characteristics, it is found that the clustering coefficient and small-world index of the functional network in PD-MCI patients were significantly lower, and the characteristic path length was significantly longer than that in the PD-nMCI group. In a topological network, the clustering coefficient reflects the local efficiency and fault tolerance (Strogatz, 2001), while short characteristic path length ensures the prompt transfers and effective integration for information between distant brain areas (Sporns and Zwi, 2004). Therefore, the decreased clustering coefficient and increased characteristic path length of functional network in PD-MCI patients suggest that in the MCI state of PD, the local and global information processing efficiency of the patient’s brain is significantly lower than that of the PD-nMCI state, and the brain’s fault tolerance rate becomes worse. In addition, there was no significant difference of topological network between the PD-nMCI and HC groups. It means that the brain topology network of early PD-nMCI patients is similar to that of normal people. When PD patients are accompanied by MCI, the topology network begins to be damaged, which also explains the susceptibility of PD-MCI in topological network.

We also used betweenness centrality from a perspective of information flow to further support the hypothesis that increased characteristic path length and decreased clustering coefficient in PD-MCI patients may be accompanied by alterations of information communication. Our results showed that the regions with significant increased nodal centrality were located in the VN, DMN, and CN, while there was decreased centrality in the SMN. The DMN and CN are two networks closely related to cognitive processes in many neurologic and psychiatric disorders, including PD, AD, depression, and autism. In particular, in studies of PD patients with MCI, one study found significantly decreased functional connectivity within the DMN in the PD-MCI group compared with that in the PD-nMCI group (Hou et al., 2016). However, another study showed that a functional disconnection of the CN could be associated with MCI in PD, rather than the DMN (Amboni et al., 2015). Additionally, a recent fMRI study found no significant differences in nodal centralities between PD-MCI and PD-nMCI, but a changing trend in the DMN, CN, and SMN (Hou et al., 2020). These similar studies show that PD-MCI patients have evidence of cognitive-related network damage, but the damage pattern still needs more research to explore. The current study also found abnormalities of nodal centralities in SMN and VN, suggesting that sensorimotor and visual-related networks may be accompanied by changes in the cognitive process of PD. Taken together, we speculate that changes in nodal centrality in the DMN, CN, SMN, and VN may be contributing factors for MCI symptoms in PD patients, which may be an important mechanism for PD-MCI patients.

It is worth mentioning that although our study found that the PD-MCI group had significant abnormalities in topological network parameters compared with the PD-nMCI group, there were relatively few patients scanned by fMRI in the database, which limited our further research. First, the gender distribution within the group is uneven, that is, the number of male patients is more than twice as high as that of female patients. Since the potential incidence of Parkinson’s disease in men is approximately twice that of women (Van Den Eeden et al., 2003), the gender distribution of groups is consistent with the incidence of Parkinson’s disease in the population, but we cannot further reduce the impact of gender differences within the group on the results. Secondly, PD-MCI patients may also be accompanied by different mental illnesses including depression, anxiety, apathy, and so on. Because the number of subjects is relatively small, it is difficult to exclude these confounding factors in our results. However, our above research results are very similar to previous studies on PD-MCI. The abnormal network nodes we found are also located in common cognitive-related networks, such as the DMN and CN. Therefore, our results based on the current sample size are interpretable, but whether PD-MCI-related topological network abnormal patterns are fixed still requires more research, a larger sample size, and better elimination of various factors for further exploration.

## Conclusion

We have investigated the topology of brain functional networks in early-stage PD patients with MCI using resting-state fMRI and graph theory analysis. Our result indicated that the brain of early-stage PD-MCI patients was related to decreased cluster coefficient, increased characteristic path length, and changed nodal centrality in the DMN, CN, SMN, and VN, which also provided a topological network insight into the role of information exchange in the underlying development of MCI symptoms in newly diagnosed PD patients.

## Data Availability Statement

The original contributions presented in the study are included in the article/Supplementary Material, further inquiries can be directed to the corresponding author/s.

## Ethics Statement

The studies involving human participants were reviewed and approved by the Shaanxi Cancer Hospital Affiliated to Xi’an Jiaotong University. The patients/participants provided their written informed consent to participate in this study.

## Author Contributions

HC proposed the study concept, designed the experiments, and modified the manuscript. XC was responsible for performing the experiments and writing the original manuscript. ML was responsible for finding relevant literature and materials, and performing the experiments. ZW was responsible for revising the manuscript. All authors contributed to the article and approved the submitted version.

## Conflict of Interest

The authors declare that the research was conducted in the absence of any commercial or financial relationships that could be construed as a potential conflict of interest.

## Footnotes

1 http://www.ppmi-info.org
