# Supplementary material for: Topological Abnormalities of Functional Brain Network in Early-Stage Parkinson’s Disease Patients With Mild Cognitive Impairment
Source: Front Neurosci. 2020 Dec 21;14:616872. doi: 10.3389/fnins.2020.616872 (PMC7793724; doi:10.3389/fnins.2020.616872)
Supplement: Supplementary file 3 [file Data_Sheet_3.DOCX]

|  | **aCp** | **aLp** | **aSigma** |
| --- | --- | --- | --- |
| **nMCI-MCI** | 0.003627 | 0.058705 | 0.078067 |
| **MCI-NC** | 0.013591 | 0.208339 | 0.642557 |
| **nMCI-NC** | 0.93016 | 0.980615 | 0.27963 |
