# Supplementary material for: Topological Abnormalities of Functional Brain Network in Early-Stage Parkinson’s Disease Patients With Mild Cognitive Impairment
Source: Front Neurosci. 2020 Dec 21;14:616872. doi: 10.3389/fnins.2020.616872 (PMC7793724; doi:10.3389/fnins.2020.616872)
Supplement: Supplementary file 5 [file Table_1.docx]

**Supplementary Table1: Functional parcellation defined by the Schaefer template with 100 parcels.**

| **ROI Label** | **ROI Name** | **ROI Network** |
| --- | --- | --- |
| **1** | 7Networks_LH_Vis_1 | VN |
| **2** | 7Networks_LH_Vis_2 | VN |
| **3** | 7Networks_LH_Vis_3 | VN |
| **4** | 7Networks_LH_Vis_4 | VN |
| **5** | 7Networks_LH_Vis_5 | VN |
| **6** | 7Networks_LH_Vis_6 | VN |
| **7** | 7Networks_LH_Vis_7 | VN |
| **8** | 7Networks_LH_Vis_8 | VN |
| **9** | 7Networks_LH_Vis_9 | VN |
| **10** | 7Networks_LH_SomMot_1 | SMN |
| **11** | 7Networks_LH_SomMot_2 | SMN |
| **12** | 7Networks_LH_SomMot_3 | SMN |
| **13** | 7Networks_LH_SomMot_4 | SMN |
| **14** | 7Networks_LH_SomMot_5 | SMN |
| **15** | 7Networks_LH_SomMot_6 | SMN |
| **16** | 7Networks_LH_DorsAttn_Post_1 | DAN |
| **17** | 7Networks_LH_DorsAttn_Post_2 | DAN |
| **18** | 7Networks_LH_DorsAttn_Post_3 | DAN |
| **19** | 7Networks_LH_DorsAttn_Post_4 | DAN |
| **20** | 7Networks_LH_DorsAttn_Post_5 | DAN |
| **21** | 7Networks_LH_DorsAttn_Post_6 | DAN |
| **22** | 7Networks_LH_DorsAttn_PrCv_1 | DAN |
| **23** | 7Networks_LH_DorsAttn_FEF_1 | DAN |
| **24** | 7Networks_LH_SalVentAttn_ParOper_1 | VAN |
| **25** | 7Networks_LH_SalVentAttn_FrOperIns_1 | VAN |
| **26** | 7Networks_LH_SalVentAttn_FrOperIns_2 | VAN |
| **27** | 7Networks_LH_SalVentAttn_PFCl_1 | VAN |
| **28** | 7Networks_LH_SalVentAttn_Med_1 | VAN |
| **29** | 7Networks_LH_SalVentAttn_Med_2 | VAN |
| **30** | 7Networks_LH_SalVentAttn_Med_3 | VAN |
| **31** | 7Networks_LH_Limbic_OFC_1 | LN |
| **32** | 7Networks_LH_Limbic_TempPole_1 | LN |
| **33** | 7Networks_LH_Limbic_TempPole_2 | LN |
| **34** | 7Networks_LH_Cont_Par_1 | CN |
| **35** | 7Networks_LH_Cont_PFCl_1 | CN |
| **36** | 7Networks_LH_Cont_pCun_1 | CN |
| **37** | 7Networks_LH_Cont_Cing_1 | CN |
| **38** | 7Networks_LH_Default_Temp_1 | DMN |
| **39** | 7Networks_LH_Default_Temp_2 | DMN |
| **40** | 7Networks_LH_Default_Par_1 | DMN |
| **41** | 7Networks_LH_Default_Par_2 | DMN |
| **42** | 7Networks_LH_Default_PFC_1 | DMN |
| **43** | 7Networks_LH_Default_PFC_2 | DMN |
| **44** | 7Networks_LH_Default_PFC_3 | DMN |
| **45** | 7Networks_LH_Default_PFC_4 | DMN |
| **46** | 7Networks_LH_Default_PFC_5 | DMN |
| **47** | 7Networks_LH_Default_PFC_6 | DMN |
| **48** | 7Networks_LH_Default_PFC_7 | DMN |
| **49** | 7Networks_LH_Default_pCunPCC_1 | DMN |
| **50** | 7Networks_LH_Default_pCunPCC_2 | DMN |
| **51** | 7Networks_RH_Vis_1 | VN |
| **52** | 7Networks_RH_Vis_2 | VN |
| **53** | 7Networks_RH_Vis_3 | VN |
| **54** | 7Networks_RH_Vis_4 | VN |
| **55** | 7Networks_RH_Vis_5 | VN |
| **56** | 7Networks_RH_Vis_6 | VN |
| **57** | 7Networks_RH_Vis_7 | VN |
| **58** | 7Networks_RH_Vis_8 | VN |
| **59** | 7Networks_RH_SomMot_1 | SMN |
| **60** | 7Networks_RH_SomMot_2 | SMN |
| **61** | 7Networks_RH_SomMot_3 | SMN |
| **62** | 7Networks_RH_SomMot_4 | SMN |
| **63** | 7Networks_RH_SomMot_5 | SMN |
| **64** | 7Networks_RH_SomMot_6 | SMN |
| **65** | 7Networks_RH_SomMot_7 | SMN |
| **66** | 7Networks_RH_SomMot_8 | SMN |
| **67** | 7Networks_RH_DorsAttn_Post_1 | DAN |
| **68** | 7Networks_RH_DorsAttn_Post_2 | DAN |
| **69** | 7Networks_RH_DorsAttn_Post_3 | DAN |
| **70** | 7Networks_RH_DorsAttn_Post_4 | DAN |
| **71** | 7Networks_RH_DorsAttn_Post_5 | DAN |
| **72** | 7Networks_RH_DorsAttn_PrCv_1 | DAN |
| **73** | 7Networks_RH_DorsAttn_FEF_1 | DAN |
| **74** | 7Networks_RH_SalVentAttn_TempOccPar_1 | VAN |
| **75** | 7Networks_RH_SalVentAttn_TempOccPar_2 | VAN |
| **76** | 7Networks_RH_SalVentAttn_FrOperIns_1 | VAN |
| **77** | 7Networks_RH_SalVentAttn_Med_1 | VAN |
| **78** | 7Networks_RH_SalVentAttn_Med_2 | VAN |
| **79** | 7Networks_RH_Limbic_OFC_1 | LN |
| **80** | 7Networks_RH_Limbic_TempPole_1 | LN |
| **81** | 7Networks_RH_Cont_Par_1 | CN |
| **82** | 7Networks_RH_Cont_Par_2 | CN |
| **83** | 7Networks_RH_Cont_PFCl_1 | CN |
| **84** | 7Networks_RH_Cont_PFCl_2 | CN |
| **85** | 7Networks_RH_Cont_PFCl_3 | CN |
| **86** | 7Networks_RH_Cont_PFCl_4 | CN |
| **87** | 7Networks_RH_Cont_Cing_1 | CN |
| **88** | 7Networks_RH_Cont_PFCmp_1 | CN |
| **89** | 7Networks_RH_Cont_pCun_1 | CN |
| **90** | 7Networks_RH_Default_Par_1 | DMN |
| **91** | 7Networks_RH_Default_Temp_1 | DMN |
| **92** | 7Networks_RH_Default_Temp_2 | DMN |
| **93** | 7Networks_RH_Default_Temp_3 | DMN |
| **94** | 7Networks_RH_Default_PFCv_1 | DMN |
| **95** | 7Networks_RH_Default_PFCv_2 | DMN |
| **96** | 7Networks_RH_Default_PFCdPFCm_1 | DMN |
| **97** | 7Networks_RH_Default_PFCdPFCm_2 | DMN |
| **98** | 7Networks_RH_Default_PFCdPFCm_3 | DMN |
| **99** | 7Networks_RH_Default_pCunPCC_1 | DMN |
| **100** | 7Networks_RH_Default_pCunPCC_2 | DMN |

**Data preprocessing**

“For the anatomical data, the T1-weighted (T1w) image was corrected for intensity non-uniformity (INU) with N4BiasFieldCorrection(Tustison et al., 2010), distributed with ANTs 2.2.0 (Avants et al., 2008), and used as T1w-reference throughout the workflow. The T1w-reference was then skull-stripped with a Nipype implementation of the antsBrainExtraction.sh workflow (from ANTs), using OASIS30ANTs as a target template. Brain tissue segmentation of cerebrospinal fluid (CSF), white matter (WM), and gray matter (GM) were performed on the brain-extracted T1w using fast FSL 5.0.9 (Zhang et al., 2001). Brain surfaces were reconstructed using recon-all from FreeSurfer 6.0.1(Dale et al., 1999), and the brain mask estimated previously was refined with a custom variation of the method to reconcile ANTs-derived and FreeSurfer-derived segmentation of the cortical gray-matter of Mindboggle (Klein et al., 2017). Volume-based spatial normalization to two standard spaces (MNI152NLin2009cAsym, MNI152NLin6Asym) was performed through nonlinear registration with antsRegistration (ANTs 2.2.0), using brain-extracted versions of both T1w reference and the T1w template. The following templates were selected for spatial normalization: ICBM 152 Nonlinear Asymmetrical template version 2009c (Fonov et al., 2009), FSL’s MNI ICBM 152 non-linear 6th Generation Asymmetric Average Brain Stereotaxic Registration Model (Evans et al., 2012).

For the functional data, the following preprocessing was performed. First, a reference volume and its skull-stripped version were generated using a custom methodology of fMRIPrep. The BOLD reference was then co-registered to the T1w reference using bbregister (FreeSurfer) which implements boundary-based registration (Greve and Fischl, 2009). Co-registration was configured with nine degrees of freedom to account for distortions remaining in the BOLD reference. Head-motion parameters with respect to the BOLD reference (transformation matrices, and six corresponding rotation and translation parameters) are estimated before any spatiotemporal filtering using mcflirt (FSL 5.0.9(Jenkinson et al., 2002)). BOLD runs were slice-time corrected using 3dTshift from AFNI 20160207 (Cox and Hyde, 1997). The BOLD time-series (including slice-timing correction when applied) were resampled onto their original, native space by applying a single, composite transform to correct for head-motion and susceptibility distortions. These resampled BOLD time-series will be referred to as preprocessed BOLD in original space, or just preprocessed BOLD. The BOLD time-series were resampled into several standard spaces, correspondingly generating the following spatially normalized, preprocessed BOLD runs: MNI152NLin2009cAsym, MNI152NLin6Asym. First, a reference volume and its skull-stripped version were generated using a custom methodology of fMRIPrep. Automatic removal of motion artifacts using independent component analysis (ICA-AROMA (Pruim et al., 2015)) was performed on the preprocessed BOLD on MNI space time-series after removal of non-steady-state volumes and spatial smoothing with anisotropic, Gaussian kernel of 6mm FWHM (full-width half-maximum). Corresponding “non-aggresively” denoised runs were produced after such smoothing. Additionally, the “aggressive” noise-regressors were collected and placed in the corresponding confounds file. Several confounding time-series were calculated based on the preprocessed BOLD: frame-wise displacement (FD), DVARS, and three region-wise global signals. FD and DVARS are calculated for each functional run, both using their implementations in Nipype (following the definitions by (Power et al., 2014)). The three global signals are extracted within the CSF, the WM, and the whole-brain masks. Additionally, a set of physiological regressors were extracted to allow for component-based noise correction (CompCor (Behzadi et al., 2007)). Principal components are estimated after high-pass filtering the preprocessed BOLD time-series (using a discrete cosine filter with 128s cut-off) for the two CompCor variants: temporal (tCompCor) and anatomical (aCompCor). tCompCor components are then calculated from the top 5% variable voxels within a mask covering the subcortical regions. This subcortical mask is obtained by heavily eroding the brain mask, which ensures it does not include cortical GM regions. For aCompCor, components are calculated within the intersection of the aforementioned mask and the union of CSF and WM masks calculated in T1w space, after their projection to the native space of each functional run (using the inverse BOLD-to-T1w transformation). Components are also calculated separately within the WM and CSF masks. For each CompCor decomposition, the k components with the largest singular values are retained, such that the retained components’ time series are sufficient to explain 50 percent of variance across the nuisance mask (CSF, WM, combined, or temporal). The remaining components are dropped from consideration. The head-motion estimates calculated in the correction step were also placed within the corresponding confounds file. The confound time series derived from head motion estimates and global signals were expanded with the inclusion of temporal derivatives and quadratic terms for each (Satterthwaite et al., 2013). Frames that exceeded a threshold of 0.5 mm FD or 1.5 standardised DVARS were annotated as motion outliers. All resamplings can be performed with a single interpolation step by composing all the pertinent transformations (i.e. head-motion transform matrices, susceptibility distortion correction when available, and co-registrations to anatomical and output spaces).”

**References:**

Avants BB, Epstein CL, Grossman M, Gee JC (2008) Symmetric diffeomorphic image registration with cross-correlation: Evaluating automated labeling of elderly and neurodegenerative brain. Med Image Anal.

Behzadi Y, Restom K, Liau J, Liu TT (2007) A component based noise correction method (CompCor) for BOLD and perfusion based fMRI. Neuroimage.

Cox RW, Hyde JS (1997) Software tools for analysis and visualization of fMRI data. NMR Biomed.

Dale AM, Fischl B, Sereno MI (1999) Cortical surface-based analysis: I. Segmentation and surface reconstruction. Neuroimage.

Evans AC, Janke AL, Collins DL, Baillet S (2012) Brain templates and atlases. Neuroimage.

Fonov V, Evans A, McKinstry R, Almli C, Collins D (2009) Unbiased nonlinear average age-appropriate brain templates from birth to adulthood. Neuroimage.

Greve DN, Fischl B (2009) Accurate and robust brain image alignment using boundary-based registration. Neuroimage.

Jenkinson M, Bannister P, Brady M, Smith S (2002) Improved optimization for the robust and accurate linear registration and motion correction of brain images. Neuroimage.

Klein A, Ghosh SS, Bao FS, Giard J, Häme Y, Stavsky E, Lee N, Rossa B, Reuter M, Chaibub Neto E, Keshavan A (2017) Mindboggling morphometry of human brains. PLoS Comput Biol.

Power JD, Mitra A, Laumann TO, Snyder AZ, Schlaggar BL, Petersen SE (2014) Methods to detect, characterize, and remove motion artifact in resting state fMRI. Neuroimage.

Pruim RHR, Mennes M, van Rooij D, Llera A, Buitelaar JK, Beckmann CF (2015) ICA-AROMA: A robust ICA-based strategy for removing motion artifacts from fMRI data. Neuroimage.

Satterthwaite TD, Elliott MA, Gerraty RT, Ruparel K, Loughead J, Calkins ME, Eickhoff SB, Hakonarson H, Gur RC, Gur RE, Wolf DH (2013) An improved framework for confound regression and filtering for control of motion artifact in the preprocessing of resting-state functional connectivity data. Neuroimage.

Tustison NJ, Avants BB, Cook PA, Zheng Y, Egan A, Yushkevich PA, Gee JC (2010) N4ITK: Improved N3 bias correction. IEEE Trans Med Imaging.

Zhang Y, Brady M, Smith S (2001) Segmentation of brain MR images through a hidden Markov random field model and the expectation-maximization algorithm. IEEE Trans Med Imaging.
