# Supplementary figures and images for: Topological Abnormalities of Functional Brain Network in Early-Stage Parkinson’s Disease Patients With Mild Cognitive Impairment
Source: Front Neurosci. 2020 Dec 21;14:616872. doi: 10.3389/fnins.2020.616872 (PMC7793724; doi:10.3389/fnins.2020.616872)

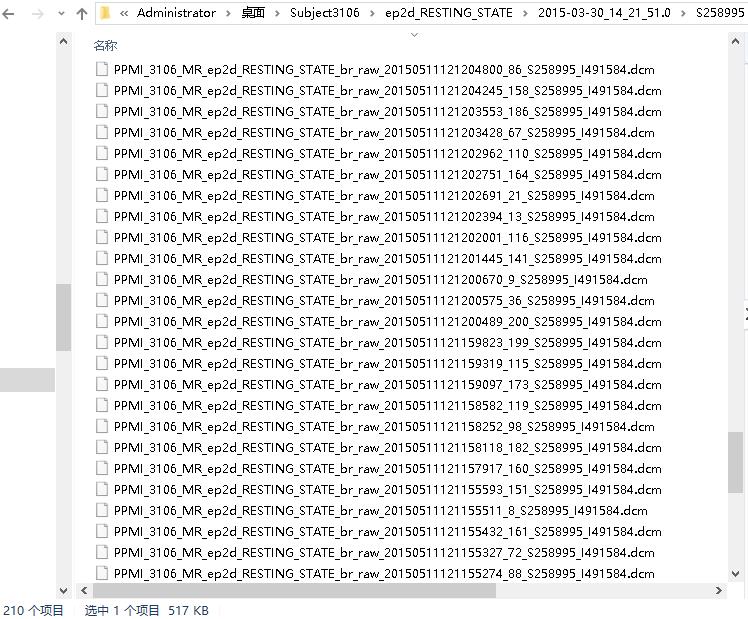

Supplement: Supplementary file 6 [file Image_1.JPEG]

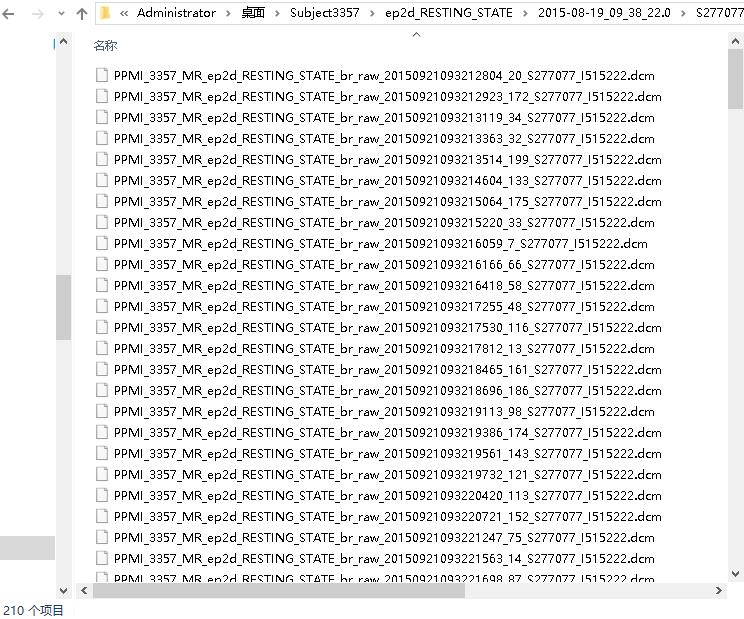

Supplement: Supplementary file 7 [file Image_2.JPEG]
